# The Power of QTL Mapping with RILs

**DOI:** 10.1371/journal.pone.0046545

**Published:** 2012-10-09

**Authors:** Shohei Takuno, Ryohei Terauchi, Hideki Innan

**Affiliations:** 1 Graduate University for Advanced Studies, Hayama, Kanagawa, Japan; 2 Iwate Biotechnology Research Center 22-174-4, Narita, Kitakami, Iwate, Japan; New Mexico State University, United States of America

## Abstract

QTL (quantitative trait loci) mapping is commonly used to identify genetic regions responsible to important phenotype variation. A common strategy of QTL mapping is to use recombinant inbred lines (RILs), which are usually established by several generations of inbreeding of an F1 population (usually up to F6 or F7 populations). As this inbreeding process involves a large amount of labor, we are particularly interested in the effect of the number of inbreeding generations on the power of QTL mapping; a part of the labor could be saved if a smaller number of inbreeding provides sufficient power. By using simulations, we investigated the performance of QTL mapping with recombinant inbred lines (RILs). As expected, we found that the power of F4 population could be almost comparable to that of F6 and F7 populations. A potential problem in using F4 population is that a large proportion of RILs are heterozygotes. We here introduced a new method to partly relax this problem. The performance of this method was verified by simulations with a wide range of parameters including the size of the segregation population, recombination rate, genome size and the density of markers. We found our method works better than the commonly used standard method especially when there are a number of heterozygous markers. Our results imply that in most cases, QTL mapping does not necessarily require RILs at F6 or F7 generations; rather, F4 (or even F3) populations would be almost as useful as F6 or F7 populations. Because the cost to establish a number of RILs for many generations is enormous, this finding will cause a reduction in the cost of QTL mapping, thereby accelerating gene mapping in many species.

## Introduction

Mapping quantitative trait loci (QTL) plays crucial roles in a number of research fields in biology. QTL mapping basically relies on detecting correlations between genetic markers and phenotypic traits in a segregating population [1]–[4]. The development of the interval mapping method [5], [6] made it possible to infer the positions of QTL with a limited number of markers. Since then, QTL mapping has been applied to various crop and vegetable species, including an early application to genome-wide QTL analysis of tomato species [7]. With the advent of molecular biology techniques such as sequencing, DNA microarray and primer extension assay [8]–[10], it became feasible to distribute a large number of markers across the genome and genotype those markers for a large sample of individuals. This revolutionary change in molecular biology further facilitated QTL mapping in many species.

Efficient fine-scale QTL mapping requires a large segregating population (bi-parental mapping population) such as an F_2_ population or Recombinant Inbred Lines (or RILs). An F_1_ population is first generated by a pair of homozygous parents (usually denoted by P_1_ and P_2_), and then selfing or sibling mating of the F_1_ individual generates an F_2_ population. It is common that each of the RILs is further selfed or sib-mated for several more generations, and F_6_∼F_7_ populations are frequently used for QTL analyses.

The advantages of using RILs for a QTL analysis are obvious. First, multiple selfing processes can increase the number of recombination events [11], which results in a finer mapping of QTLs. More importantly, once RILs are established, in which the genotypes of all lines are fixed as homozygotes, these lines can be repeatedly used for investigating QTLs of various phenotypes under different environments. Thus, the establishment of a comprehensive set of RILs will be a substantial contribution to QTL mapping of the species.

In the meantime, QTL mapping is frequently applied to species that do not have substantial resources at the single-lab level. In this case, it is not reasonable to establish comprehensive RILs; rather, it makes more sense to conventionally map a rough location of a QTL with a limited amount of effort. There is an obvious tradeoff between the performance of QTL mapping and the cost required, including the sample size and the number of generations of selfing or sibling mating. The heaviest labor would be to maintain a number of RILs for multiple generations, so that a simple idea is to use a younger generation with a limited number of RILs. As a consequence, as a most aggressive setting, there are a number of QTL mapping studies that conventionally used an F_2_ population.

In such a small-scale QTL mapping, it is very useful if we have some ideas about the relationship between the performance (statistical power) and the cost (the number of selfing or sibling mating generations, sample size, and marker density), which will greatly help to optimize the design of the QTL mapping experiment. This problem has been extensively investigated in simple theoretical models [12]–[14]. Here, we provide the results of extensive simulations in more realistic situations. We assume that a large number of markers are distributed across the genome, and that they are partially linked. With these results, we discuss how the cost can be reduced by minimizing the reduction of the performance.

## Methods

### Model and simulation

For simulating QTL mapping process with RILs, we consider a diploid species. It is assumed that the genome consists of 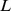 chromosomes with equal lengths and that the genome size is 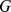 Mb, which corresponds to 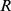 centimorgan (cM). It is also assumed that 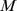 markers are evenly distributed across the genome. We set a single QTL in the simulated genome, and ask whether we can find significant phenotype-genotype correlations for markers nearby the QTL. To assess the performance of QTL mapping, we simulate the process of creating a large number of RILs from a single pair of parental lines, P_1_ and P_2_, both of which are assumed to be completely homozygote. Their hybrid progeny, F_1_, is created, and then 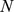 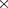
F_2_ progenies are produced by selfing F_1_. It is assumed that each of the F_2_ progenies is successfully inbred by the singe-seed-descent method for six generations (*i.e.*, up to F_7_). Throughout this process, recombination occurs randomly at rate 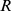, following the four-strand model [15]. It is also assumed that at least one chiasma form in each chromosome in one meiosis event, called obligate chiasma [16]–[18], but for simplicity, we also assume no crossing-over interference. At each generation from F_2_ to F_7_, a simple QTL mapping method (see below) is applied.

In the QTL mapping process, it is assumed that all markers are genotyped for all individuals, and the phenotype of each individual is determined by a simple model, in which there is a particular locus that partially contribute to the quantitative trait of interest [1]–[5]. Let 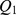 and 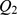 be the two alternative alleles at this QTL inherited from the two parental lines (P_1_ and P_2_). Then, it is assumed that the phenotype of each diploid individual in the segregating population is determined by the genotype at this locus. There are three possible states, 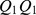, 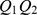, 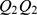, which are denoted by genotypes 1, 2, and 3, respectively. The numbers of individuals with the three genotypes are denoted by 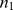, 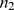, and 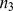, and 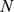 is the total number of individuals (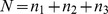).

Let 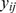 be the quantitative value representing the focal phenotype of the 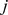
th individual in the 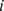
th genotype (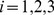 and 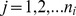), then in a simple model with no interaction between genotypes and environment, 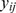 can be written as
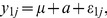
(1)

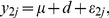
(2)

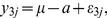
(3)where 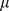 is the mid-parental value, 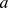 is the additive genetic effect and 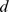 is the dominance effect. Other factors are represented by 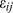, including the environmental variance and the residual genotypic variance due to other unlinked QTLs. 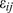 is assumed to follow a normal distribution with mean 0 and variance 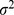. We assume that this factor 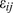 is added at each generation independently. In other words, only 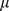, 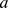 and 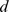 are the parameters that determine the genetic factors that can be inherited through generations, and 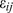 is not affected by the phenotype or genotype at the previous generation. Simulations of RILs under this simple model are used for investigating the performance of QTL mapping. Assuming a large number of markers are available across the genome, we simply perform a statistical test of the null hypothesis of no association between the phenotype and each of all markers. We do not need to use the interval mapping method because of the availability of a large number of markers (this condition will be relaxed later). We use two likelihood ratio tests to examine if there is a significant phenotype-genotype correlation.

In the first method (Method I), if 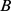 and 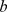 represent the two alleles from P_1_ and P_2_, respectively, the null model assumes equal average phenotypes of the three genotypes, 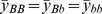. Alternatively, if the marker and the QTL is completely linked, we expect 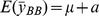, 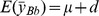 and 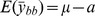. Method I requires the likelihoods of the observation (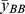, 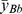, 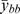) under these two extreme cases (null and alternative). It should be noted that this very commonly used method requires estimation of the dominance effect (*e.g.*, [6], [19], [20]). Alternatively, the second method, which we propose here, is a simplified version (Method II), in which only homozygote individuals with marker genotypes, 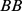 and 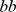 are considered (heterozygotes, 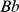, are excluded), and tests the null hypothesis of 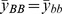. We propose this conventional method because it does have to involve the dominance parameter by excluding heterozygotes from the analysis. Estimation of the dominance parameter has to rely on a relatively small number of heterozygotes, which will likely cause a great deal of uncertainty in the estimate. We suspected that miss-inference of the dominance parameter due to such uncertainty might result in a reduction of the power. Obviously, the situation would be identical when selfing generations increase and all RILs become homozygote in the entire genome. The two methods are described below in detail.

#### Method I

This method involves computation of the maximum likelihoods of the observation, (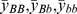), under the null and alternative models. The latter involves maximum likelihood estimation of the four unknown parameters, 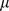, 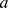, 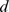, and 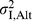, which are given by
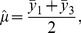
(4)

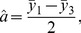
(5)

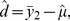
(6)and
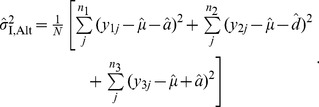
(7)Then, it can be considered that the maximum likelihood of the observation under the alternative scenario is given these estimates. That is, the log-maximum likelihood is computed by
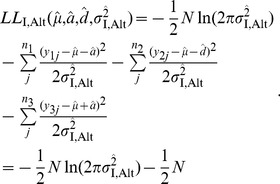
(8)


In the null model, in which only two parameters (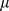 and 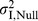) are involved, the maximum log-likelihood of the data is given by
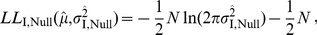
(9)where 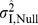 is simply given by
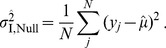
(10)

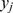
 represents the phenotypic value of the 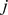
th individual (with no specification of genotype, so that 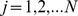).

Thus, the maximum log-likelihoods under the null and alternative models are computed by equations (9) and (8), respectively, from which the LOD score can be obtained by 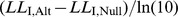. For each replication of the simulations, we set a cut-off value of the LOD score by 1,000 replications of a permutation test [21], so that the false positive rate is set at 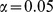 after correcting for multiple testing by multiplying the *P*-value by the number of markers (*i.e.*, Bonferroni correction). Note that because a permutation test is performed for each data set, the false positive rate is always 5% for any parameter set in all generations. This allows a fair comparison of the performances of different models with different parameters.

#### Method II

This method is a simplified version of Method I, in which marker-heterozygous individuals are excluded so that it does not involve the process of estimating the dominance parameter. In the alternative model of Method II, 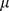 and 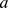 can be estimated from the average phenotypes, 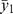 and 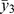:
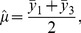
(11)

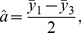
(12)and 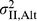 is given by
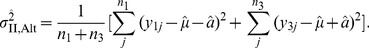
(13)This process is basically identical to that for Method I. Then, the maximum likelihood of the observation under the alternative scenario is given with these given these estimates:
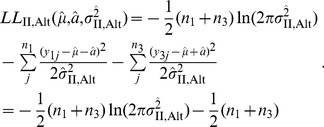
(14)


In the null model, where only two parameters (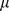 and 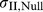) are involved as well as Method I, the maximum log-likelihood of the data is given by
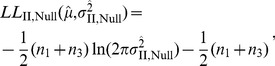
(15)where 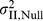 is simply given by
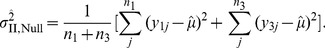
(16)Then, from equations (14) and (15), the LOD score can be computed as 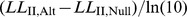.

## Results

### Simulation results

We designed simulations to quantitatively evaluated the effect of the number of generations on the performance of QTL mapping. Throughout this article, we fix 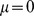 and 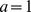. We assume a simple model, in which the simulated genome consists of 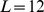 chromosomes with equal length 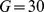 Mb, so that the genome size (360 Mb) is similar to that of rice, a species to which QTL mapping is frequently applied. In total 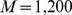 codominant DNA markers are evenly distributed on the genome, such that the interval length is 300 kb (100 markers per chromosome). The recombination rate is assumed to be 4 cM/Mb, which is roughly consistent with estimates of rice [22]. Some of these simulation conditions will be relaxed later.

We are interested in the power of QTL mapping to detect a particular QTL that has a significant genetic contribution. It is assumed that this QTL locates at the center of one chromosome. This location is also the middle of two adjacent markers; therefore, the distance to the closest marker is 150 kb. Although the model does not set other specific QTLs, their effect is incorporated in the environmental factor, 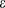 in equations (1–3). For each of these parameter settings, we performed 10,000 independent replications of simulations from F_1_ to F_7_, and at each generation (except for F_1_) the LOD scores were computed for all markers.

**Figure 1:**
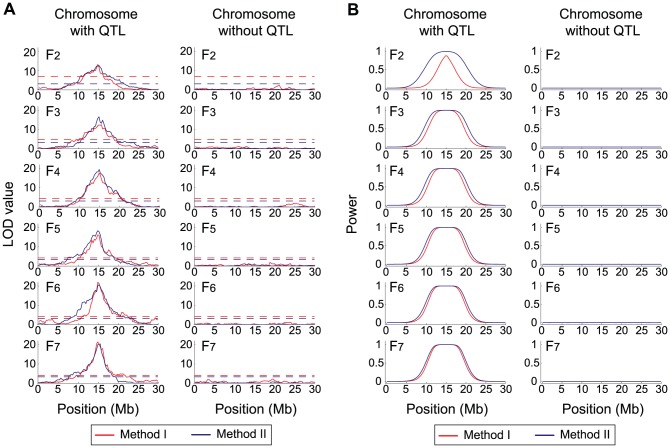
Evaluating the power of QTL mapping by simulations. **a** The distributions of the LOD values at markers along chromosomes (left: the chromosome with the QTL, right: a chromosome representing the other chromosomes without the QTL). The QTL is located at the middle of the chromosome (left panel). The red and blue lines show the LOD scores of Method I and Method II, respectively. The result is from a single replication of the simulation with 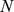 = 200, 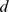 = 0, and 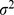 = 2. The 5% cutoff values for the two methods are shown by broken lines. **b** The distributions of the power of the two methods, which were obtained by 10,000 replications.

A typical pattern of the results is shown in Figure 1, in which 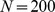, 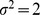, and no dominance (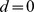) were assumed. The expected heritability in the F_2_ population is given by
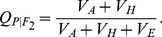
(17)Therefore, with this parameter set, we expect that the expected heritability is 20% (note that the heritability changes in the following F_3_, F_4_,… generations). It was found that on the chromosome with the QTL (left panel in Figure 1A), both Methods I and II provide the highest LOD score around the QTL, creating a sharp peak, whereas the LOD scores on all other chromosomes are low (plot for one representative chromosome is shown in the right panel in Figure 1a). We confirmed that similar patterns hold for all simulated parameter sets unless 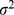 is very large.

We found that there are at least two notable observations in Figure 1. (i) The distributions of LOD scores do not change much through generations, suggesting that significant power of detecting QTL may be expected even in early generations. If so, QTL mapping does not necessarily require many generations of inbreeding, so that a huge amount of time and cost could be saved. (ii) The performance of Method II exceeds that of Method I in many cases, especially at early generations. Method II is a simplified method that does not use heterozygous markers, whereas Method I uses all samples. It is suggested that the simpler method without considering the dominance effect (Method II) may be more efficient even with an obvious drawback of reducing sample size. These two observations have significant implications that F_3_∼F_4_ populations could have reasonable power for QTL mapping and that Method II would perform better at such early generations.

In order to quantitatively evaluate these hypotheses, we investigated the power of QTL mapping. The right panel of Figure 1 summarizes the results of 10,000 replications of the simulations with the same parameters as those used for the left panel. The power was computed for each SNPs, which is defined as the proportion of the replications, in which the LOD score is significant at the 5% level (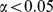, after correcting for multiple testing). The spatial distributions of the power support our two hypotheses; the performance of Method II (blue line) overall exceeds that of Method I (red line) and the power at F_4_ is almost comparable to that at F_7_.

**Figure 2:**
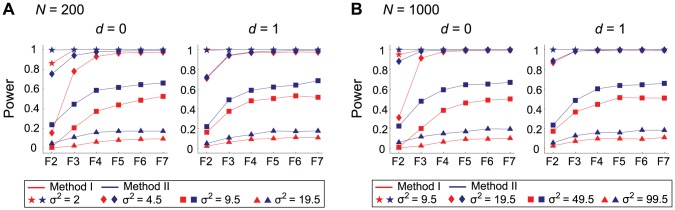
The power of QTL mapping as a function of the number of inbreeding generations. **a** 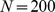 and **b** 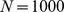 are assumed. The red and blue lines are for the results of Method I and Method II, respectively. See text for details.

Further simulations with wide ranges of parameters were carried out to confirm if this holds. The results are summarized in Figure 2. In this figure, we mainly focus on how the environmental variance (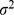) affects the power in two sample sizes, 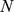 = 200 and 1,000. We also considered two cases: no dominance (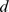 = 0) and complete dominance (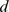 = 1). We used a wide range of 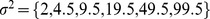 (the corresponding heritability at the F_2_ generation are 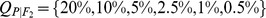), and partial results are shown in Figure 2 such that the power at F_7_ distributes roughly from 0.1 to 1. The power is here defined as the proportion of simulation replications in which the LOD scores of both of the two closest makers to the QTL are significant at the 5% level (after correcting for multiple testing). As the power is overall much higher when 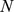 = 1,000, we found that the QTL can be detected with probability 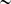
1 when 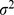 is smaller than 9.5 (that is, larger heritability; Figure 2B), while the QTL with 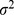 = 9.5 would be detected with probability roughly 0.5 when 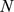 = 200 (Figure 2A).

These simulations supported that our two hypotheses hold with these wide ranges of parameters. For all the parameter sets, the performance of Method II exceeds that of Method I especially at early generations and the power of Method II at F_4_ is almost comparable to that of F_7_. These seem to be true regardless of the degree of dominance. It should be noted that as mentioned earlier, the power is measured by a permutation applied to each data set, so that the false positive rate is alway controlled to be 5% for all parameter sets. Therefore, the comparison of power is statistically fair.

**Figure 3:**
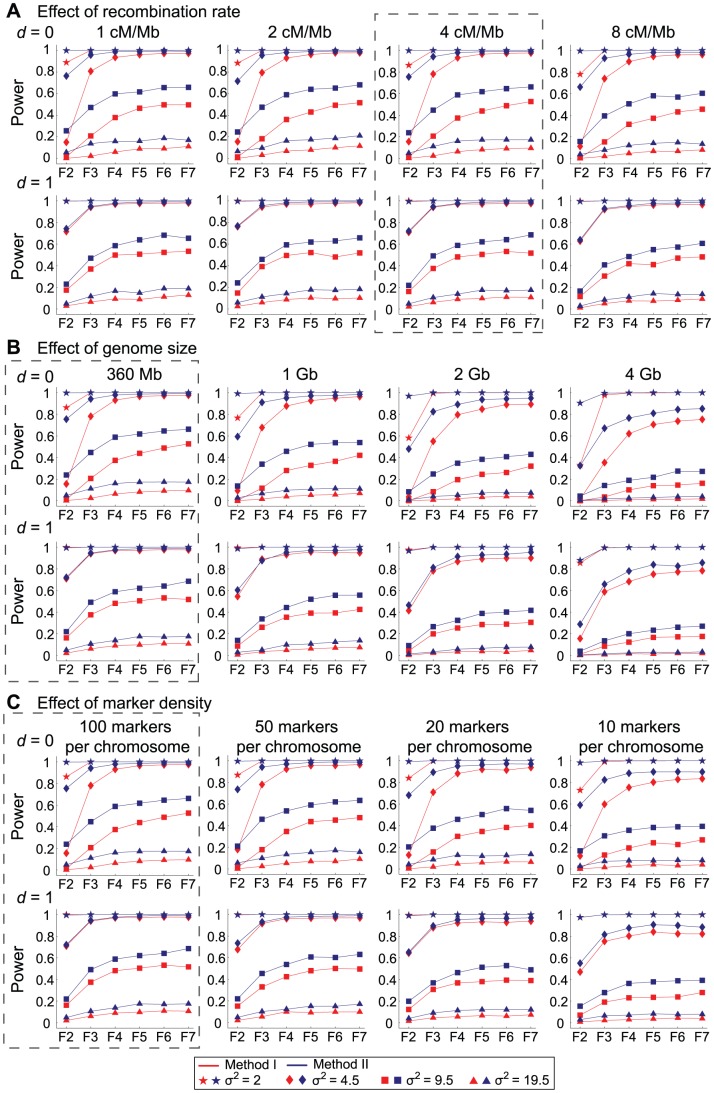
The effects of recombination rate (a), genome size (b) and marker density (c) on the power of QTL mapping. The panels in broken squares are identical to Fig. 2a.

In Figure 3, we investigated the effects of other parameters including the recombination rate, genome size, and marker density. It is found that overall the effects of these parameters are small. In Figure 3A, the power is shown for the recombination rate is changes from 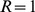 to 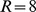, while all other parameters remained the same as those used for Figures 1 and 2A. The panel in the broken square is identical to Figure 2A. In Figure 3B, the effect of genome size is investigated. Because our initial setting may be applied to species with small genomes such as Arabidopsis and rice, the genome size is increased up to 4 Gb, which is almost as large as maize and wheat. In Figure 3C, the marker density is reduced to up to 10 times. We found that the overall patterns are similar to one another, although the power becomes relatively weak when marker density is low (the leftmost panel in Figure 3C, and also see the leftmost panel of Figure 3B). There also seems to be a weak negative correlation between the power and the recombination rate (Figure 2A). Thus, our conclusion could be robust to these parameters.

**Figure 4:**
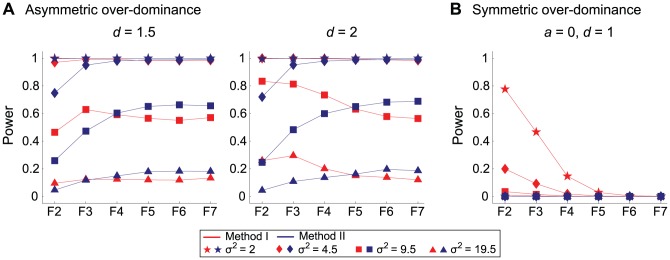
The power of QTL mapping with over-dominance. (**a**) Asymmetric over-dominance. (**b**) Symmetric over-dominance. See text for details.

These results are for the cases of relatively normal settings with additive phenotype effect at the focal QTL. However, there are cases where this does not obviously hold. One example is over-dominance. Suppose the phenotypic value of heterozygote individuals at the focal QTL are expected to be larger than those of homozygotes. Such a situation can be realized by setting 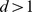, so that the expected phenotype value for 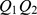 heterozygotes exceeds that of 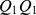 homozygotes (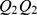 homozygotes always have smallest values. See equations (1–3)). To investigate the power of the two methods under this setting, we repeated the same power simulations by assuming 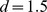 and 2. (we don't need to mention 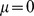, 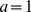 and 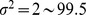 if they are identical to those above.) With these settings, because the phenotype of heterozygotes are very informative to identify the QTL, the overall performance of Method I is quite good (Fig. 4A). This is remarkable especially in earlier generations, but the situation becomes similar to those with the QTL with the additive phenotype effect as the number of generation increases because almost all individuals become homozygotes. This pattern is remarkable in the extreme case, symmetric overdominance, where 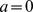 is given so that the expected phenotype values of 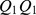 and 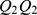 homozygotes are identical and the phenotype of heterozygotes exceeds homozygotes by 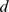 (Fig. 4B). In earlier generations, Method I works fairly well, but the power is almost zero in F_6_ and F_7_ because almost all individuals are homozygotes, either 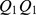 and 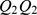, between which there is no difference in phenotype.

### Linked QTLs

We also consider a more complicated model, in which there are QTLs that are linked to the focal QTL. It should be noted that our basic model described above takes into account the effect of multiple QTLs, whose effects are included in the third term of the right-hand side of equations (1–3). The assumption was that those QTLs are not linked to the focal QTL. We here investigate the effect of linked QTLs to the focal QTL.

We use a simple two-locus model. The alleles from P_1_ at the two loci are denoted by Q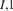 and Q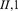 and those from P_2_ are denoted by Q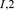 and Q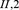. 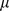, 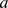 and 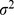 were set such that their 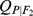 are 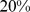 and 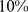, respectively, in the codominance case. Other parameters follow those used in the earlier simulations for Figure 2A. These two QTL are linked, and four different distances between them were considered (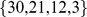 Mb). No epistasis between QTLs was assumed.

**Figure 5:**
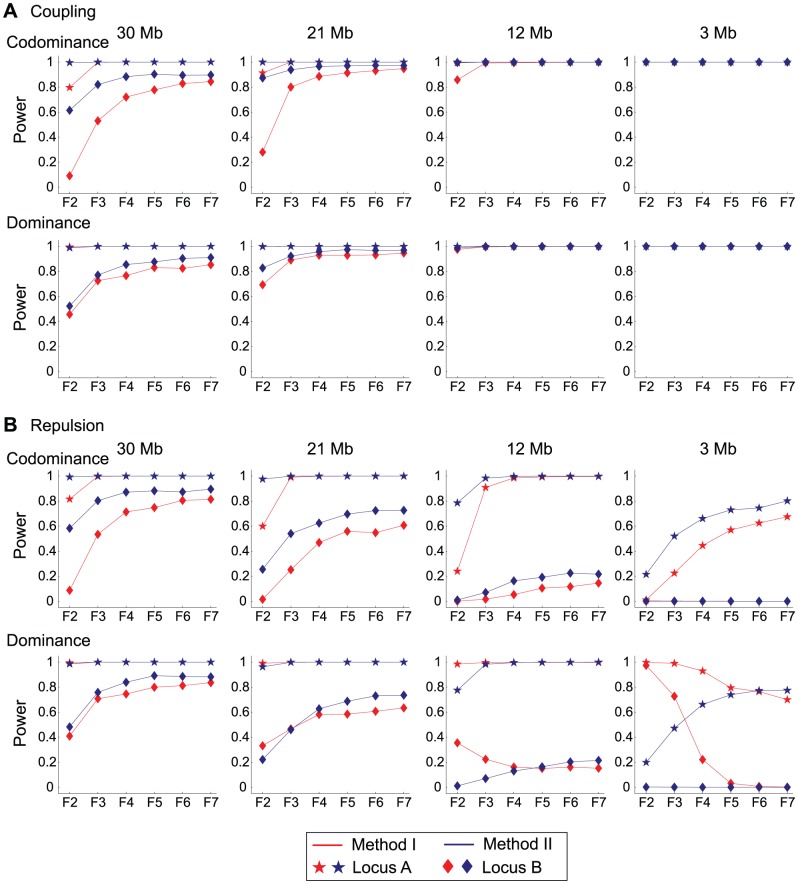
The power to detect two linked QTLs under the two-locus model. (**a**) Results when the two QTLs have a coupling effect. (**b**) Results when the two QTLs have a decoupling effect. The power is shown for each QTL. See text for details.

We first consider the cases of coupling phenotype effect, that is, both of the two alleles from P_1_ (*i.e.*, Q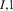 and Q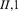) have positive effects on the phenotype. The results are summarized in Figure 5A, which shows the power to detect each QTL in the codominance and dominance cases. The overall patterns are quit similar to each other. When the distance is short (12 and 3 Mb), we observe very high power because the two QTLs behave almost as a single QTL with relative contribution 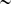
30%. As the distance increases, the power decreases because of recombination. If the distance is significantly long (*i.e.*, 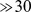 Mb), the two QTLs behave almost independently, so that the power to detect them should become comparable to those shown in Figure 2A. The performance of Method II is better than Method I in all cases.

Figure 5B shows the power when the phenotype effects of the two QTLs are decoupling or repulsion, that is, Q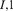 and Q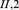 have positive effects on the phenotype. We first consider the codominance case. Because alleles with positive and negative effects are initially coupled, the power is much more lower than in the case of coupling (Figure 5A). Recombination between the two QTLs creates coupling haplotypes, Q
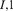
Q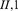 and Q
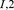
Q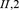, thereby increasing the power. Indeed, the power increases with increasing the number of generations and the distance between the QTLs. The performance of Method II is overall better than Method I.

The pattern is more complicated in the dominance case. With few recombinations (*i.e.*, in younger generations with short distance), heterozyge individuals have the largest phenotypic values, so that they are very informative. This is why we observe higher performance of Method I. When the distance is 3 Mb, the power of Method I in F_2_ is almost one because of the striking difference between homozygotes and heterozygotes. As more recombination events occur, the advantage of Method I is getting smaller, and the pattern becomes similar to the codominance case.

Thus, when there are multiple QTLs especially with dominance effect and/or epistatic interaction, the relationship between the phenotype parameters (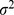) and the power is complicated. In such a case, it is quite common that we observe a single peak of high LOD scores encompassing the two QTLs. In a practical case, the problem would be that it is very difficult to know whether a single peak of the LOD score involves only one QTL or multiple QTLs. To distinguish these cases, further breeding should be required. For example, see refs. [23], [24].

## Discussion

QTL mapping plays significant roles to identify genetic regions responsible to important phenotype variation. One of the common strategies of QTL mapping uses a large number of RILs, which are established for at least several generations of inbreeding (typically up to F_6_ or F_7_). We here used simulations to quantitatively evaluate the performance of QTL mapping using RILs. Under the simple model with one focal QTL, it was found that the performance of QTL mapping with F_4_ population could be almost comparable to that with F_6_ or F_7_ populations (Figures 2 and 3). It was also found that Method II has more power than Method I especially at earlier generations. Method II is a simplified version of Method I, and it does not involve the process to estimate the dominance parameter, 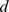. An obvious drawback of Method II is a reduction of sample size because it discards marker-heterozygote samples. For example, roughly 25% and 12.5% of RILs are excluded at F_3_ and F_4_, respectively. Nevertheless, the performance of Method II exceeds that of Method I, suggesting that the uncertainty of 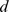 might reduce the power of Method I. Thus, our results imply that QTL mapping does not necessarily requires RILs at F_6_ or F_7_ generations; rather, F_4_ (or even F_3_) populations would be almost as useful as F_6_ or F_7_ populations. Although we quit the simulations at F_7_, it is expected that the results for further generations can be intuitively understood; Because the power is almost saturated at F_6_∼F_7_ for many parameter sets, the power for F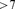 cannot be much larger than that of F_7_. Only when the power is still increasing at F_7_, more power is expected for F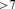, but it would eventually saturate in a few generations. Soller and Beckmann [12] suggested relatively little gain of the power by increasing the number of inbreeding generations when heritability is large, based on their theoretical analysis under a two-locus model (*i.e.*, QTL vs. marker). Our simulations support their implication in more practical situations with a number of markers for a wide range of 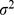. While we only simulated RILs with selfing, these conclusions should hold for RILs with sibling mating, which was confirmed by a limited amount of additional simulations. We found that the only effect of sibling mating is that the decrease of heterozygous loci is slightly retarded (data not shown).

Further simulations under various conditions were performed (Figure 3) to investigate the effects of the parameters that were fixed in the basic simulation for Figures 2 and 3. The investigated parameters are the recombination rate, genome size, and marker density, while the sample size was fixed to be 200. It was found that these factors have relatively minor effects on the results, indicating that our conclusions should hold under wide ranges of the parameters. It was surprising that the power did not decrease much when we have only 10 markers on a 30 Mb (120 cM) of chromosome. An implication is that in order to reduce the cost, a reasonable level of power could be expected when there are roughly every 10 cM.

In contrast, it seems that the effect of the sample size is much larger than those of the factors explored in Figure 3. As shown in Figure 2, QTLs with much larger 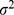 can be detected when 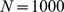 in comparison with the case of 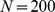. Increasing sample size is costly, may be as much as extending inbreeding generations, but our results imply that the former may be more efficient than the latter. We would suggest that increasing the sample size is one of the best strategies to improve the performance rather than continuing inbreeding for many generations. Because the cost to establish a number of RILs for many generations is enormous, it is important to understand the relationship between the cost and output. Our results provide several ideas to obtain better performance with a limited cost, there by accelerating gene mapping in many species.

In summary, we demonstrated that our idea of ignoring heterozygotes (incorporated in Method II) works quite well in a relatively simple situations. The major difference between the two methods is that Method I has an additional parameter (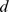) that has to be estimated from data. Our demonstration might indicate that simple methods with no estimation process work well. In this sense, one might think that a linear regression analysis might also work well [13], [14]. However, although this analysis does not involve estimation of the dominance parameter, it assumes a certain level of dominance (most commonly no dominance). Therefore, when the true dominance parameter is different from the assumption, the power might be reduced. In other words, it still involves uncertainty of the dominance parameter. As expected, we confirmed that the performance of the linear regression analysis did not exceed that of Method II for all parameter range (data not shown). Our Method II provides a general framework in evaluating likelihood ignoring heterozygote. This can be readily incorporated in the interval mapping method [1]–[6], or recently developed more computationally sophisticated QTL mapping algorithms, such as, Baysian shrinking method e.g., [25], [26] and penalized maximum likelihood e.g., [27].

We mainly obtained these conclusions under a simple model with one focal QTL, but they can be applied to broad cases because the model does not necessarily assumes that there is only one QTL in the genome. We simply focused on a single QTL with its phenotype effect specified by parameter 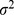 (the effects of other QTLs are included in the environmental factors, 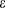, in equations 1–3). Therefore, as long as the focal QTL is not linked to other QTLs, our conclusions should hold. We confirmed this by additional simulations in a model allowing multiple QLTs with various quantitative effects, although too obvious theoretically.

It should be noted that there are some cases where the performance of Method I exceeds that of Method II, as demonstrated in Figures 4 and 5. The consensus of these cases is that the phenotype of heterozygotes is informative. One is the case of overdomenace, where the performance of Method I is much better in earlier generations because there are a number of heterozygotes. The situation is similar when there are two linked QTLs that have decoupling phenotype effects with complete dominance. Also in this case, the phenotype value of double heterozygotes is the highest, Method I performs well particularly in earlier generations. We should keep in our mind that our major conclusions may not hold in these cases (may not be very common though).

## Conclusions

QTL mapping plays significant roles to identify genetic regions responsible to important phenotype variation. One of the common strategies of QTL mapping uses a large number of RILs, which are established for at least several generations of inbreeding (typically up to F_6_ or F_7_). We here used simulations to quantitatively evaluate the performance of QTL mapping using RILs. It was found that the performance of QTL mapping with F_4_ population could be almost comparable to that with F_6_ or F_7_ populations (Figs. 2 and 3). It was also found that Method II has more power than Method I especially at earlier generations. Method II is a simplified version of Method I, and it does not involve the process to estimate the dominance parameter, 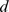. An obvious drawback of Method II is a reduction of sample size because it discards marker-heterozygote samples. For example, roughly 25% and 12.5% of RILs are excluded at F_3_ and F_4_, respectively. Nevertheless, the performance of Method II exceeds that of Method I, suggesting that the uncertainty of 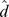 might reduce the power of Method I. Thus, our results imply that in most cases, QTL mapping may not necessarily require RILs at F_6_ or F_7_ generations; rather, F_4_ (or even F_3_) populations would be almost as useful as F_6_ or F_7_ populations. Because the cost to establish a number of RILs for many generations is enormous, this finding will cause a reduction in the cost of QTL mapping, thereby accelerating gene mapping in many species.

## References

[1] Weir BS, Eisen EJ, Goodman MM, Namkoong G (1987) Proceedings of the Second International Conference on Quantitative Genetics. Sunderland, MA: Sinauer Associates.

[2] Tanksley SD (1993) Mapping polygenes. Annu Rev Genet 27: 205–233. 8122902 10.1146/annurev.ge.27.120193.001225

[3] Falconer DS, Mackay TFC (1996) Introduction to Quantitative Genetics, Ed 4. Harlow, Essex, UK: Longmans Green.

[4] Lynch M, Walsh JB (1998) Genetics and Analysis of Quantitative Traits. Sunderland, MA: Sinauer Associates.

[5] Lander ES, Botstein D (1986) Mapping complex genetic traits in human: New methods using a complete RFLP linkage map. Cold Spring Harbor Symp on Quant Biol 51: 49–62. 10.1101/sqb.1986.051.01.007 2884068

[6] Lander ES, Botstein D (1989) Mapping mendelian factors underlying quantitative traits using RFLP linkage maps. Genetics 121: 185–199. 2563713 10.1093/genetics/121.1.185 PMC1203601

[7] Paterson AH, Lander ES, Hewitt JD, Peterson S, Lincoln SE, et al (1988) Resolution of quantitative traits into Mendelian factors by using a complete linkage map of restriction fragment length polymorphisms. Nature 335: 721–726. 2902517 10.1038/335721a0

[8] Singer T, Fan Y, Chang HS, Zhu T, Hazen SP, et al (2006) A high-resolution map of Arabidopsis recombinant inbred lines by whole-genome exon array hybridization. PLoS Genet 2: e144. 17044735 10.1371/journal.pgen.0020144 PMC1564425

[9] Paterson AH (2006) Leafing through the genomes of our major crop plants: strategies for capturing unique information. Nat Rev Genet 7: 174–184. 16485017 10.1038/nrg1806

[10] Gupta PK, Rustgi S, Mir RR (2008) Array-based high-throughput DNA markers for cropimprovement. Heredity 101: 5–18. 18461083 10.1038/hdy.2008.35

[11] Jansen RC (2003) Quantitative trait loci in inbred lines. In: Balding DJ, Bishop M, Cannings C, editors. Handbook of Statistical Genetics. Chichester, UK: John Wiley & Sons. 589–618.

[12] Soller M, Beckmann JS (1990) Marker-based mapping of quantitative trait loci using replicated progenies. Theor Appl Genet 80: 205–208. 24220897 10.1007/BF00224388

[13] Hu Z, Xu S (2008) A simple method for calculating the statistical power for detecting a QTL located in a marker interval. Heredity 101: 48–52. 18446184 10.1038/hdy.2008.25

[14] Kao CH, Zeng MH (2010) An investigation of the power for separating closely linked QTL in experimental populations. Genet Res (Camb) 92: 283–294. 20943009 10.1017/S0016672310000273

[15] Emerson S (1969) Linkage and recombination at the chromosome level. In: Caspari EW, Ravin AW, editors. Genetic Organization. New York & London: Academic Press. 267–360.

[16] Haldane J (1931) The cytological basis of genetical interference. Cytologia 3: 54–65.

[17] Mather K (1937) The determination of position in crossing-over. II. The chromosome lengthchiasma frequency relation. Cytologia Fujii Jubilee Vol: 514–526.

[18] Henderson SA (1963) Chiasma distribution at diplotene in a locust. Heredity 18: 173–190.

[19] Haley CS, Knott SA (1992) A simple regression method for mapping quantitative trait loci in line crosses using flanking markers. Heredity 69: 315–324. 16718932 10.1038/hdy.1992.131

[20] Hayashi T, Ukai Y (1994) Detection of additive and dominance effects of QTLs in interval mapping of F2 RFLP data. Theor Appl Genet 87: 1021–1027. 24190538 10.1007/BF00225798

[21] Churchill GA, Doerge RW (1994) Empirical threshold values for quantitative trait mapping. Genetics 138: 963–971. 7851788 10.1093/genetics/138.3.963 PMC1206241

[22] International Rice Genome Sequencing Project (2005) The map-based sequence of the rice genome. Nature 436: 793–800. 16100779 10.1038/nature03895

[23] Zhang L, Li H, Li Z, Wang J (2008) Interactions between markers can be caused by the dominance effect of quantitative trait loci. Genetics 180: 1177–1190. 18780741 10.1534/genetics.108.092122 PMC2567366

[24] Li H, Hearne S, Bänziger M, Li Z, Wang J (2010) Statistical properties of qtl linkage mapping in biparental genetic populations. Heredity 105: 257–267. 20461101 10.1038/hdy.2010.56

[25] Meuwissen TH, Hayes BJ, Goddard ME (2001) Prediction of total genetic value using genome-wide dense marker maps. Genetics 157: 1819–1829. 11290733 10.1093/genetics/157.4.1819 PMC1461589

[26] Xu S (2003) Estimating polygenic effects using markers of the entire genome. Genetics 163: 789–801. 12618414 10.1093/genetics/163.2.789 PMC1462468

[27] Zhang YM, Xu S (2005) A penalized maximum likelihood method for estimating epistatic effects of QTL. Heredity 95: 96–104. 15931238 10.1038/sj.hdy.6800702

